# The Incidental Influence of Memories of Past Eating Occasions on Consumers’ Emotional Responses to Food and Food-Related Behaviors

**DOI:** 10.3389/fpsyg.2016.00943

**Published:** 2016-06-21

**Authors:** Betina Piqueras-Fiszman, Sara R. Jaeger

**Affiliations:** ^1^Marketing and Consumer Behaviour Group, Department of Social Sciences, Wageningen UniversityWageningen, Netherlands; ^2^The New Zealand Institute for Plant & Food Research LimitedAuckland, New Zealand

**Keywords:** consumer research, emotions, recalled meals, memory priming, affect infusion, food, overeating

## Abstract

Our memories of past eating experiences are influential in shaping food preferences and consumption behavior, and the emotions that people associate to these memories are linked to their attitudes toward foods and their everyday food-related behaviors. This work studies the impact that food-related memories have on peoples’ emotional state and how this state is projected in a subsequent evaluation of images pertaining to food and food-related behaviors. Focus is placed on guilt and shame emotions. Through an online survey, three memories were investigated (a positive meal, a routine evening meal, and an overeating occasion) among UK consumers (*N* = 710). Participants primed with the overeating memory evaluated images related to junk food as conveying more feelings of guilt and shame than did participants primed with the memory of a positive meal. Moreover, this effect was moderated by participants’ dietary restraint status. Participants classified as having a high dietary restraint had stronger associations with the emotions guilt and shame than participants classified as low in dietary restraint. In contrast, a memory of a positive meal did not lead to positive valuations of any of the food-related images shown. Overall, the findings from the present study illustrate the partial impact that personal food memories have on consumers’ emotional response toward food-related issues, which in turn has the potential to affect future behavior. This study therefore contributes to the literature about cognitive effects on food attitudes and behavior. Furthermore, the results suggest that the empirical approach may be tapping into possibly unconscious emotions toward foods and food-related behavior.

## Introduction

The measurement of consumers’ emotion-related responses toward foods and beverages has been intensively explored in recent years (see Köster and Mojet, 2015; Meiselman, 2015 for reviews). The premise driving this activity is that emotions and moods play an important role in consumer behavior (Gardner, 1985) at the point of purchase and in the subsequent consumer–product relationship (Batra et al., 2012). The influence of mood on eating behavior and the influence of food on mood and emotions are underpinned by complex relationships involving physiological factors, emotional coping mechanisms, eating tendencies/personality traits, sociological factors, psychological factors, expectations based on previous experiences, and memory (Köster and Mojet, 2015).

The experience of the consequences of past eating can significantly impact subsequent eating behavior since the integration of internal and external signals that influence food intake relies on memory systems (Booth, 1992). For example, Robinson et al. (2011, 2012) showed that how we recall food enjoyment can influence subsequent enjoyment of foods. Two studies examined whether remembered enjoyment of eating a food can be increased and whether this makes individuals more likely to eat that food in the future. A simple manipulation of instructing participants to revisit what they found enjoyable about a food immediately after eating it was used to increase remembered enjoyment (relative to controls). In a separate study, they explored whether the manipulation to increase remembered enjoyment resulted in participants choosing to eat more of a food as part of a later buffet lunch. Both hypotheses were confirmed. Taken together, these findings suggest that remembered enjoyment can be modified by simply recalling a past occasion, resulting in an increase or decrease in the amount of food chosen and eaten later. Moreover, recent evidence suggests that episodic memory of food, or thoughts of future meals, suppresses subsequent food intake (Vartanian et al., 2016).

In light of the strong interplay between food enjoyment, emotions, and memories (e.g., Philippot and Schaefer, 2001), Piqueras-Fiszman and Jaeger (2015a,b) investigated the emotions associated to experienced meals recalled as being memorable and the individual differences explaining this relationship. In (online) studies, respondents described either one particular memorable meal or a routine evening meal as if they were telling to a friend and then indicated the strength of association to 40 emotions when thinking about the meal. The emotion profile of a recalled routine evening meal followed the same pattern as that for a recalled memorable meal, but the routine character was reflected on specific emotions (e.g., less exciting and more boring). Importantly, these patterns varied across different age groups, genders, and respondents’ trait emotional character. For instance, on average, men and older consumers had more positive emotional associations, which may be related to life experiences and gender roles. Participants who scored higher on trait positive emotional intensity, or food involvement, had, on average, more positive emotional associations to recalled meals than people who were less intense in these personality traits.

A particular relevant area of research in which food behavior is closely linked with emotions is that of dietary goal-conflict and coping mechanisms. Although the majority of the research in this area focuses on cohorts with eating disorders (e.g., binge eating, depressive symptoms; Frank, 1991; Skinner et al., 2012), overeating has been found to be relatively regular in a large portion of many populations (Steenhuis, 2009) and highly linked to emotionally driven eating (Davis et al., 2007). The most commonly studied discrete emotions in this context are shame and guilt, as they are potential predictors of coping responses in weight-related behavior (Conradt et al., 2008). For instance, it has been shown that people with goal-conflicts or eating disorders experience significantly higher levels of shame and guilt in relation to eating than normal eaters do (e.g., Frank, 1991).

In the current study, we extend research on meal/food memorability (Piqueras-Fiszman and Jaeger, 2015a,b) by further exploring people’s emotional response to different types of food-related memories (i.e., how certain memories change our emotional state). Focus was directed to the incidental impact of those memories on how people emotionally respond to food-related actions and objects. Moreover, we particularly delve into the effect of an overeating memory on guilt and shame considering the impact of dietary restraint on emotional responses.

The underlying principle of the present approach can be explained by cognitive-priming theories, particularly the affect infusion model (AIM; Forgas, 1995), whereby affectively loaded information exerts an influence on, and becomes incorporated into, the evaluation process and eventually distorting the outcome. These are instances, for example, in which a person’s affect elicited by one stimulus/event (e.g., the emotion evoked by a food stimulus) infuses judgments of another, unrelated target stimulus (e.g., images of other people’s faces; Keltner and Lerner, 2010). Past studies have shown that people tend to find others more attractive when they feel good (Griffitt, 1970; Gouaux, 1971; Gouaux and Summers, 1973; Clark and Waddell, 1983); judge others as more aggressive when they feel fear (Feshbach and Singer, 1957); and interpret facial expressions (Schiffenbauer, 1974), social events, or even behaviors (Forgas et al., 1984), and make choices (Garg et al., 2005) according to prior emotions.

When judgments require a degree of constructive processing, people may use either a substantive or a heuristic processing strategy to arrive at an outcome. The AIM further identifies two alternative mechanisms of affect infusion: affect-priming and affect-as-information, likely to operate during substantive and heuristic processing, respectively. According to the affect-priming principle, affect may indirectly influence judgments during substantive processing through its selective influence on attention, encoding, retrieval, and associative processes (Clark and Waddell, 1983; Forgas and Bower, 1987; Singer and Salovey, 1988; Bower, 1991). On the other hand, the affect-as-information principle states that feelings can directly inform judgments during fast, heuristic processing as people use their affective state as a shortcut to infer their evaluative reactions to a target.

In the present study, we built on cognitive-priming theories, particularly the AIM, to explore people’s emotional response to different types of food-related memories and the impact of those memories on how people emotionally respond to food-related actions and items. For this purpose, a two-stage empirical approach was implemented, whereby people were firstly asked to recall a food-related event and in a subsequent unrelated task, they evaluated images depicting food or food-related behavior. Three different emotion-inducing memory priming conditions were considered. These aimed to cover possible events that people might recall with some frequency and that might influence their attitudes and behavior toward food (e.g., recalling a negative event might make a person feel anxious and wanting to eat; Raghunathan and Pham, 1999; Garg et al., 2007). Participants were asked to remember either a positive meal experience, an everyday routine evening meal, or an occasion when they had mindlessly overeaten. The two first memory priming conditions have been shown to induce a general positive emotional state (Piqueras-Fiszman and Jaeger, 2015a,b). The third memory priming condition, as mentioned earlier, was deemed relevant for its link to weight-related coping mechanisms. In addition, this condition is more emotion-specific (eliciting shame/guilt) and thus it is expected that its effects depart from what one would predict based on emotional valence (eliciting a generally negative emotion state). Images were used as target stimuli for ease of data collection and represented a range of food stimuli (items and actions) that people encounter in their daily life. That is, the focus of the target stimuli was the general category and what it conveyed, rather than the specific details displayed in each image. To the best of our knowledge, little research has been conducted investigating how people’s own memories of past food-related events incidentally affect their emotional response to foods and food-related behaviors, and particularly on how memories of overeating elicit shame and guilt. This, in turn, could trigger particular future behaviors, such as more mindful eating (Higgs, 2008).

According to the AIM, people’s emotional state influences the quality of their impression formation judgments (Forgas, 2015). Hence, participants would tend to evaluate the images in accordance with their prevailing emotional state (assimilation effect). This leads to our first two hypotheses. The first hypothesis (H1) stated that the positive memorable meal would lead to stronger positive emotional evaluations of the images compared to the other two memory priming conditions.

In alignment with H1, but related to negatively valenced emotions, the second hypothesis (H2a) was that the overeating memory priming condition would elicit stronger negative emotional evaluations that the other two conditions. Considering that shame and guilt are known to be coping responses to goal-conflict, H2b stated that, additionally to this general negativity infusion, feelings of shame and guilt would be prominent when evaluating images depicting high-caloric foods or someone eating these foods (vs. the other images of healthier foods or actions).

A secondary aim of the present research was to study the moderating effect of individual differences in eating restraint on emotional responding. Goal-conflict is stronger among restrained eaters and direct emotion responses to food have been shown to be dependent on this trait (e.g., Van Gucht et al., 2014). Therefore, our third hypothesis (H3a) stated that that highly restrained participants would associate stronger feelings of guilt and shame to the unhealthiest food-related images than unrestrained participants, and that (H3b) this difference would be larger in the overeating memory condition.

## Pre-Test and Main Study Base-Line/Manipulation Check Data

### Pre-test to Select Images for the Main Study

#### Rationale

Although the main focus of the research was not the images of food items/actions themselves, but the incidental effect of memory priming on their evaluation, care needed to be taken with selection of target images for the main study and three criteria were considered. The first required the images to be clearly recognizable and understood by participants since incidental perceptual cues (e.g., high/low visibility of image content) has been shown to hinder ease of processing, which in turn may impact on impressions formed about the inferred affective characteristics of the target images (Forgas, 2015). In addition, the images also had to convey the expected emotions and be easily understood. In relation to the second criterion, the images needed to be balanced in terms of the emotions they conveyed (valence and arousal). For instance, a univalenced image set (i.e., displaying mainly positive or negative content) could lead to valence habituation among participants, which in turn, could influence their responses and the possible effect of the memory primes. The third criterion sought to eliminate images that evoked intense emotional responses. This criterion was enforced to enable the memory priming conditions to influence the emotions participants experience when viewing the images.

The criteria above were applied to the target food item/action images, as well as non-food “distractor” images. It was decided to include these to avoid making the aim of the study too obvious and biasing participants’ responses. A total of 28 images (target and filler) were sourced from the internet (no copyright violations), and categorized by the researchers as spanning a two-dimensional emotional space (valence and arousal). The images represented objects (e.g., a burning tree, a cute puppy, and a mixed green salad) and people engaging in different actions (e.g., people in a rollercoaster, an obese person eating junk food, and children fighting). Images that showed faces of people in detail were purposefully avoided since when making complex kinds of social judgments it is likely to find various motivational, cultural, and normative influences superimposed on the purely cognitive processes assumed by models such as the AIM. All images were shown in full color and in the same size (250 × 200 pixels).

Data collection was done online and a survey hosted by a professional marketing research agency (3GEM, UK) was completed by 450 adult UK residents balanced in terms of gender and age (19–34, 35–49, or 50–70 years old). Section “Participants” describes participant inclusion/exclusion criteria, which were identical in this pre-test and the main study.

#### Image Pre-test: Procedures for Data Collection and Analysis

The 28 images included in the pre-test were assessed in terms of the emotional associations they conveyed by means of two direct measurement approaches. This was done to achieve independence from the data collection method, and a total of 300 participants completed a verbal task and 150 completed a pictorial task (with very similar, balanced, and demographic characteristics).

The verbal emotion task was based on the emotion affective circumplex model (Feldman Barrett and Russell, 1998), which proposes that a person’s subjective experience of emotion is a cognitive interpretation of the neurophysiological experience of valence and arousal in a given situational context (Russell, 2003). The emotion words, which spanned the two-dimensional space defined by a valence axis and an arousal axis, were: *relax, content, happiness, joy, enthusiasm, worry, disappointment, frustration, sadness, boredom*, and *surprise*. To manage the respondent burden, only 11 emotion terms were included. Responses were obtained using the bulls-eye approach which shows a target with 10 concentric circles, to make it more engaging for respondents. The instructions given to participants were: “Please rate the feelings each picture conveys by dragging the terms into the target (1–10 scale). The closer a term is placed to the center of the target, the stronger it conveys the feeling. Place a term at the outermost circle of the target when the picture does not convey the feeling.” Since focus was directed to what emotions the images were perceived as conveying rather than how the participants felt when looking at the images, the emotion words were presented as nouns and not as adjectives (e.g., happiness vs. happy and frustration vs. frustrated). Across participants, the emotion words were presented in random order. Images were shown sequentially. To avoid respondent fatigue, participants evaluated 14 of the 28 images (balanced in terms of emotion valence) in random order (i.e., images were evaluated the same number of times as number of participants, 150).

The pictorial emotion task implemented the self-assessment Manikin (SAM; Lang, 1980), commonly used in the cognitive psychology literature, for the dimensions of pleasure/valence and arousal. Along each of the two dimensions, five simple humanoid figures depicting values on a continuously varying scale is used to indicate emotional reactions. One SAM scale ranges from a smiling, happy figure to a frowning, unhappy figure when representing the valence dimension. For the arousal dimension, the other SAM scale ranges from an excited, wide-eyed figure to a relaxed, sleepy figure. These scales were presented in fixed order with the valence scale preceding the arousal scale. Respondents were asked to rate the feeling that a picture conveyed by selecting any of the five figures comprising each SAM scale, or between any two figures, which results in a nine-point rating scale for each dimension, and an example was provided. Each participant rated all 28 images (shown sequentially) on the two SAM scales. The order of images was randomized across participants.

One-way analyses of variance (ANOVAs) were performed on the ratings of the 11 emotion words (verbal task) and the SAM valence and arousal data (pictorial task) considering the type of image as independent variable. Multiple comparisons were performed with Tukey’s test (α = 0.05). The analyses were performed using XLStat 2015 (Addinsoft, New York, NY, USA).

#### Pre-test Results: Images Selected for Inclusion in the Main Study

Following ANOVA on the data of the 28 images, 10 images were selected for inclusion in the main study. This was a suitable number to ensure an array of images (six targets and four distractors) without making the study too burdensome for respondents. The target six images selected for inclusion in the main study, which represented more vs. less healthy foods and two food-related behaviors, are referred to as: “mixed salad,” “roast chicken,” “junk food,” “burnt food,” “movie and popcorn,” and “obese and junk food” (see Supplementary Material). The mean scores for each of the 11 emotion words and the two SAM scales confirmed that none of the 10 images conveyed extremely intense emotions (**Supplementary Material, Table S1**). In all instances, ratings for the food-related images were between 1.6 and 7.5 of 10. This satisfied the third criterion for image selection described in Section “Rationale for Pre-Test to Select Images for Main Study.”

### Base-Line Data for Images Used in the Main Study

**FIGURE 1:**
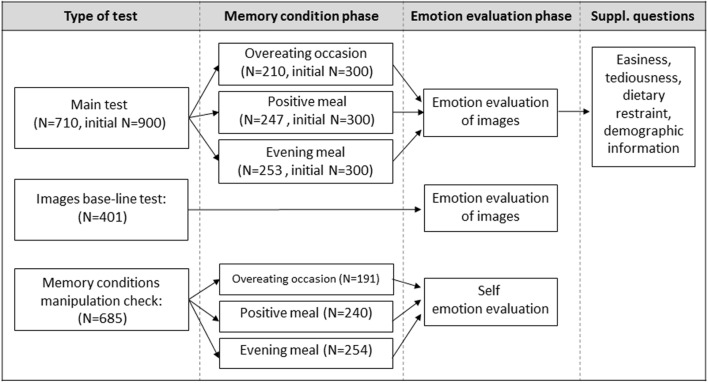
**Diagram of the procedure flow for the main test and parallel tests conducted to obtain base-line measurements of the images and the memory conditions.** The samples of each test are independent.

Base-line data for the images, when evaluated without a prior memory priming task, were obtained and were necessary to infer that results from the main study deviating greatly from the “image alone” values were due to memory priming (**Figure 1**).

A total of 401 UK adult participants (56% female; 19–70 years old, *M* = 42, *SD* = 13) assessed the 10 images using the bulls-eye protocol from the image pre-test. A revised list of 18 emotions was used, identical to the main study (listed and explained in Section “Supplementary Questions”). Responses were collected via an online survey hosted by 3GEM (UK) and participants were randomly drawn from the same sample population as those taking part in the main study (same inclusion/exclusion criteria). For clarity of communication, details are presented here and not as part of the main study.

One-way ANOVA was conducted on the 18 emotion terms (dependent variables) considering the image as independent variable. The data confirmed that the 10 selected images evoked the expected emotional associations (**Supplementary Material, Table S2**). For instance, positive emotions (*N* = 9) were rated higher for the image of “roast chicken” than the image of “burnt food” (3–4 points difference of 10). Similarly, negative emotions (*N* = 8) were rated higher for the image of “obese and junk food” than “movie and popcorn” (2–3 points difference of 10). Expected differences were also seen for specific emotions. For example, the “burnt food” image was rated higher in *frustration* than the “movie and popcorn” image (*M* = 6.1 vs. 2.3) and for *nostalgia* the mean ratings were higher for the “roast chicken” image than for the “mixed salad” image and “obese and junk food” images (*M* = 5.3 vs. 3.3).

### Manipulation Check for Memory Primes Used in the Main Study

Also for experimental rigor, confirmation was needed that the memory primes evoked the expected emotional associations and that these were different for the three recalled meals (a positive meal, an evening meal, and a meal where they had overeaten).

A total of 685 UK adults took part (55% female; 19–70 years old, *M* = 43, *SD* = 14), evenly and randomly distributed across the three memory conditions (**Figure 1**). The instructions were, respectively: “Try to recall as vividly as possible [a past specific eating experience that you are fond of/one of your usual weekday evening dinner occasions/a past specific occasion after having mindlessly eaten much more than intended on your own]. Please describe this […] in 5–10 short sentences. Imagine you were telling a good friend/family member about it.” Using the bulls-eye protocol from the pre-test, participants were asked “How does thinking about this memory make you feel?” and responded by dragging into the target each of the 18 emotion terms (identical to those in the main study). Responses were collected via an online survey hosted by 3GEM (UK) and participants were randomly drawn from the same sample population as those taking part in the main study (same inclusion/exclusion criteria). Thus, data collection occurred in parallel with the main study, but, similar to above, is reported here for clarity of communication.

One-way ANOVAs were conducted on the 18 emotion terms (dependent variables) considering the memory priming condition as independent variable. It was expected based on Piqueras-Fiszman and Jaeger (2015a,b) that emotions evoked by the positive meal and evening meal memory primes would be dominantly positive. This was established (**Table 1**) and it was also found, as expected, that the positive meal condition evoked stronger positive emotional associations than the routine evening meal condition. In both instances, the strength of association to negative emotions was weak (∼2 of 10). The emotional responses from participants who were asked to recall a meal where they had overeaten was less positive and more negative, with a tendency for some negative emotions to be more strongly associated than positive emotions (e.g., *guilt* and *shame*) as expected. Although this was the memory prime that evoked the most intense negative emotions (mostly for *guilt*, *M* = 6.2) the mean values remained moderate (4–5 of 10; *sadness*, *frustration*, *tension*, *disappointment*). Against this background, the overeating condition was only moderately negative, and, at the same time, moderately positive. Conversely, the positive meal experience was genuinely positive, as evidenced by its mean values.

**Table 1:** Results from the main study manipulation check for memory priming conditions (i.e., without image evaluation) showing mean ratings of the emotion terms reported directly from the three memory condition groups: positive meal, evening meal, and overeating occasion (*N* = 685).

Emotions terms	Positive meal		Evening meal		Overeating				
						
	*M*	*SD*	*M*	*SD*	*M*	*SD*	*F*_(2,682)_	*p*	η^2^
Warm	8.18c	2.21	6.77b	2.92	4.21a	3.03	113.78	<0.0001	0.25
Shame	1.73a	1.76	1.93a	2.15	5.44b	3.40	148.86	<0.0001	0.30
Boredom	1.83a	1.72	2.59b	2.55	3.66c	2.87	30.87	<0.0001	0.08
Relax	7.36c	2.59	6.71b	2.87	4.45a	2.87	62.67	<0.0001	0.16
Courage	3.76b	3.09	3.07a	2.67	2.94a	2.61	5.53	0.004	0.02
Disappointment	1.74a	1.80	2.20a	2.20	5.12b	3.45	110.84	<0.0001	0.25
Enthusiasm	7.03c	2.64	5.67b	3.03	3.70a	2.78	74.12	<0.0001	0.18
Content	7.72c	2.62	6.80b	3.01	4.72a	3.05	59.07	<0.0001	0.15
Guilt	2.04a	2.06	2.30a	2.29	6.17b	3.29	170.13	<0.0001	0.33
Frustration	2.09a	2.11	2.25a	2.23	4.55b	3.16	63.77	<0.0001	0.16
Joy	7.87c	2.43	6.24b	3.01	4.08a	3.09	94.42	<0.0001	0.22
Happiness	8.61c	1.92	7.02b	2.87	4.78a	3.04	112.73	<0.0001	0.25
Worry	2.12a	2.23	2.21a	2.35	4.35b	3.20	49.33	<0.0001	0.13
Nostalgia	5.99b	3.19	4.39a	3.09	3.97a	2.89	27.03	<0.0001	0.07
Surprise	4.31b	3.29	3.21a	2.73	3.88ab	2.90	8.59	<0.0001	0.02
Pride	6.13b	3.12	5.61b	3.15	3.01a	2.70	63.27	<0.0001	0.16
Sadness	1.96a	2.15	2.22a	2.22	4.18b	2.84	51.69	<0.0001	0.13
Tension	2.02a	2.21	2.27a	2.92	3.86b	3.03	36.35	<0.0001	0.10


*Emotion responses obtained using a bulls-eye approach where 1 = “Not felt” and 10 = “Felt very strongly.” Means with differing letters within rows are significantly different at the *P* < 0.05 based on Tukey’s HSD paired comparisons. Sample sizes of conditions: Evening meal *N* = 254; Positive meal *N* = 240; Overeating occasion *N* = 191.*

## Main Study Methodology

As previously described, the primary aim of this research was to better understand how memory priming incidentally influences consumers’ emotional associations to food and food-related actions. In relation to this general aim, prior to image evaluations, respondents completed a memory priming task wherein they recalled one of three past occasions (a memorable meal, a routine evening meal, and a meal where they had overeaten). This is a well-established methodology in the affect domain to induce emotions (e.g., Lerner and Keltner, 2001). The moderating effect of restrained eating on image evaluation was also considered (secondary research aim).

### Participants

A total of 900 adult UK residents participated (who had not taken part in pre-tests or manipulation checks). The exclusion criteria were: being allergic to any kind of food or having any eating disorders (to avoid these factors strongly biasing the responses), and educational attainment less than college (to avoid potential low quality responses). The main study was hosted by the research provider used for the pre-test (3GEM, UK) and similar to the pre-test, base-line data collection, and manipulation checks, participants completed the survey from a private location. Participants were balanced in terms of age (19–34, 35–49, or 50–70 years old) and gender across the three memory priming conditions.

### Memory Priming and Emotional Associations Evoked by Images

At the start of the survey, participants were told that they would be asked to complete two different tasks. The complete procedure is shown in **Figure 1.** The first of these was the memory priming task. There were three memory priming conditions labeled for simplicity: positive meal, evening meal, and overeating, and participants were randomly assigned only to one of them. The instructions were, respectively: “Try to recall as vividly as possible [a past specific eating experience that you are fond of/one of your usual weekday evening dinner occasions/a past specific occasion after having mindlessly eaten much more than intended on your own]. Please describe this […] in 5–10 short sentences. Imagine you were telling a good friend/family member about it.” The vividness with which participants recalled their memory was self-reported on a seven-point scale (1 = “not at all vivid” to 7 = “very vivid”).

Following the memory priming task, respondents continued with an unrelated task, which consisted of evaluating the 10 selected images in terms of the emotions they evoked. Respondents therefore would make no connection with the memory task, and focus was directed to what emotions the images were perceived as conveying rather than how the participants felt when looking at the images. This was important to avoid self-reflection, which might lead to somewhat unnatural results. All the images were presented sequentially in random order and with instructions that were identical to those used in the verbal pre-test (Section “Image Pre-Test: Procedures for Data Collection and Analysis”), participants were asked to drag listed emotions/feelings into the bullseye target (1–10 scale).

Seeking to better capture emotional associations jointly evoked by the memory conditioning task and the target images, the list of emotion words was expanded from that used in the image pre-test. In the main study, a total of 18 emotion words were used, and these were selected by the researchers from previous studies (Richins, 1997; Piqueras-Fiszman and Jaeger, 2015a,b) and from the pre-test, and based on the relevance to possibly describe how they felt after recalling the different memories while covering the affective space: *warm-heartedness, courage, enthusiasm, content, joy, happiness, pride, relax, nostalgia, surprise, disappointment, guilt, shame, frustration, boredom, worry, sadness*, and *tension*. Nine have a general positive connotation, eight a negative connotation, and *surprise* can be either positive or negative depending on the context.

Immediately following the emotion task, participants were asked to answer two questions on seven-point scales: (1) “How easy was it for you to complete this task?” and (2) “How tedious was it for you to complete this task?”

### Supplementary Questions

To address the second aim, participants’ level of dietary restraint was approximated using questions from the Dutch eating behavior questionnaire (DEBQ: Van Strien et al., 1986). For the purpose of keeping the questionnaire short, only two items were used: “Do you take into account your weight with what you eat?” and “Do you deliberately eat less in order not to become heavier?” (five-point scale: never, seldom, sometimes, often, and very often).

Questions about respondents’ demographic information, household structure, and annual household income formed the last section of the survey, together with a space for them to add any thoughts they had about the tasks.

### Data Analyses

Prior to analysis, data were discarded if participants had completed the survey in less than 10 min (estimated minimum time to properly answer the questionnaire), and if the answers seemed to have been provided randomly or unthoughtfully. It was considered important that participants had vividly evoked the focal memories of their past meals, and participants who stated that the memory was “not at all vivid” (one of seven) were also discarded.

After the deletions, the data of 710 respondents of 900 were retained for input to analysis (55% female; 19–70 years old, *M* = 43, *SD* = 13) and the proportions across the memory priming conditions were: positive meal: 35%, evening meal: 37%; and overeating: 28%. In these data, the mean vividness scores for the recalled meals (i.e., the three memory priming conditions) were high (*M* = 5.5, *SD* = 1.3), and the positive meal was recalled significantly more vividly than the other two (*M* = 5.9 vs. 5.3, *P* < 0.001). Furthermore, it was established that no significant differences between the three memory priming conditions existed with regard to perception of the emotion task (*P* > 0.05). This was perceived as quite easy (*M* = 5.4, *SD* = 1.6), although somewhat tedious (*M* = 4.7, *SD* = 1.8), which likely could be linked to the repetitive aspect of the task whereby each of the 10 images had to be rated for each of the 18 emotion words.

All analyses were conducted for each image separately (i.e., one for each of the six food-related images: “mixed salad,” “roast chicken,” “junk food,” “burnt food,” “movie and popcorn,” and “obese and junk food”). To address H1 (the positive memorable meal would lead to stronger positive emotional evaluations of the images compared to the other two conditions), ratings for the nine positive emotions were averaged and an ANOVA was conducted on the resulting average (dependent variable) considering the type of memory condition (positive meal, overeating, or evening meal) as independent variable. The data were averaged since when inspecting the patterns of all the nine discrete emotions, the three conditions followed the same pattern across them. Thus, reporting and analyzing an average simplifies the description without missing important information for the research questions established.

To address H2a (the overeating condition would elicit stronger negative emotional evaluations that the other two conditions), ratings for the eight negative emotions were averaged and an ANOVA was conducted on the resulting average (one dependent variable) considering the type of memory condition as independent variable. H2b stated that the overeating condition would particularly elicit *shame* and *guilt* when evaluating images depicting high-caloric foods or someone eating these foods. Therefore, another ANOVA was conducted only on the ratings of *guilt* and *shame* (two dependent variables) considering the memory condition as independent variable.

Finally, to address H3a (highly restrained participants would associate stronger *guilt* and *shame* to the unhealthiest food-related images than unrestrained participants), and that this difference would be larger in the overeating memory condition (H3b), an ANOVA was conducted on the *guilt* and *shame* ratings (two dependent variables) considering memory condition, the level of dietary restraint and their interaction as independent variables.

Regarding participants’ self-reported dietary restraint and the moderating influence on image evaluation, the two DEBQ items were highly correlated (*r* = 0.69, *P* < 0.0001; *M* = 3.0, *SD* = 1.1). Using scores averaged across the two items, participants were divided in two similar-sized groups (median split with cut-off value = 3.00; low – L: *N* = 371; *M* = 2.2, *SD* = 0.7; high – H: *N* = 339; *M* = 3.8, *SD* = 0.7). We opted for this dichotomizing approach since our aim was not to investigate this effect, and the interaction with the memory condition, in detail across all levels of dietary restraint. For our research question, creating two groups (higher and lower) suffice to be able to explore the effect of dietary restraint.

Multiple comparisons for the emotion data were performed with Bonferroni correction, for other data Tukey’s HSD test was used. The data analyses were performed using XLStat 2015 (Addinsoft).

## Main Study Results

### Effects of Food-Related Memory Priming on Positive Emotional Associations to Images of Food and Food-Related Behaviors

The first hypothesis stated that the positive memorable meal would lead to stronger positive emotional evaluations of the images compared to the other two memory conditions (H1). The emotion ratings of the nine positive emotions were averaged and the ANOVA results revealed that there was no significant effect of the memory condition on the average score of the positive emotions in none of the six images. **Table 2** shows the average ratings of the positive emotions for each image and the ANOVA results, where it can be seen that the Positive meal memory did not lead to higher average positive emotional state compared to the other two conditions. H1 is therefore rejected.

**Table 2:** Results pertaining to Hypothesis 1. Means, standard deviations, and ANOVA results on the effect of memory condition on the average rating of the positive emotions.

Images	Evening meal		Positive meal		Overeating			
					
	*M*	*SD*	*M*	*SD*	*M*	*SD*	*F*_(2,707)_	*P*
Burning food	2.50	1.70	2.76	1.93	2.79	1.91	1.81	0.164
Chicken roast	5.39	2.23	5.60	2.08	5.65	2.12	0.98	0.377
Junk food	4.13	2.22	4.28	2.19	4.08	2.28	0.51	0.602
Movie and popcorn	4.66	2.03	4.96	2.02	4.74	2.03	1.42	0.242
Obese and Fries	2.72	1.89	2.80	1.87	2.86	1.85	0.30	0.738
Mixed salad	4.59	2.25	4.79	2.24	5.06	2.22	2.57	0.077


*1 = “Not felt” and 10 = “Felt very strongly.” Sample sizes of conditions: evening meal *N* = 253; positive meal *N* = 247; overeating occasion *N* = 210.*

### Effects of Food-Related Memory Priming on Negative Emotional Associations to Images of Food and Food-Related Behaviors

When inspecting the average negative emotion score, two generally noticeable results emerges. One is that the image “obese and junk food” contributed in evoking the highest negative score in the three conditions. The second was that, where significant effects were found, these were caused by the overeating condition resulting in the highest scores (except for the image “mixed salad,” where those in the positive meal condition scored highest).

H2a stated that the overeating memory condition would elicit stronger negative emotional evaluations that the other two conditions. The results partly support this hypothesis (**Table 3**). The overeating occasion condition lead to a significantly stronger negative (average) emotion for all images except for “burning food” image (no difference among the three conditions) and the image “mixed salad” (for which the positive meal condition lead to the highest average). The overeating occasion memory lead to a significantly higher negative emotion average than the other two conditions only when evaluating the “junk food” image (*M*_O_ = 3.86, *SD*_O_ = 1.88 vs. *M*_E_ = 3.10, *SD*_E_ = 1.87, *P* < 0.0001, *d* = 0.38, vs. *M*_P_ = 3.32, *SD*_P_ = 1.97, *P* = 0.012, *d* = 0.26). For the other three images, the negative emotion average was significantly higher than the evening meal, but not more than the positive meal.

**Table 3:** Results pertaining to Hypothesis 2. Means, standard deviations, and ANOVA results on the effect of memory condition on the average score of the negative emotions, guilt, and shame.

Images	Evening meal		Positive meal		Overeating					
							
	*M*	*SD*	*M*	*SD*	*M*	*SD*	*F*_(2,707)_	*P*	η^2^	*d*^∗^
**Average**
Burning food	4.72a	2.17	4.90a	2.13	4.92a	2.20	0.67	0.511	0.01	–
Chicken roast	2.26a	1.36	2.55ab	1.70	2.64b	1.70	3.57	0.029	0.01	0.24
Junk food	3.10a	1.87	3.32a	1.97	3.86b	2.18	8.65	0.000	0.03	0.38/0.26
Movie and popcorn	2.66a	1.62	2.86ab	1.81	3.08b	1.88	3.28	0.038	0.01	0.24
Obese and fries	4.59a	2.20	4.88ab	2.13	5.26b	2.15	5.52	0.004	0.02	0.31
Mixed salad	2.20a	1.45	2.67b	1.89	2.48ab	1.72	4.91	0.008	0.02	0.28
**Shame**
Burning food	4.74a	3.30	4.92a	3.34	4.90a	3.27	0.226	0.797	0	–
Chicken roast	1.87a	1.78	2.13a	2.14	2.18a	2.12	1.687	0.186	0	–
Junk food	3.38a	2.91	3.66a	2.90	4.36b	3.25	6.334	0.002	0.02	0.32/0.23
Movie and popcorn	2.34a	2.25	2.50a	2.33	3.13b	2.74	6.610	0.001	0.02	0.32/0.25
Obese and fries	5.44a	3.41	6.00ab	3.17	6.51b	3.31	6.038	0.003	0.02	0.32
Mixed salad	1.79a	1.55	2.23b	2.14	2.11ab	2.07	3.548	0.029	0.01	0.23
**Guilt**
Burning food	4.33a	3.35	4.45a	3.03	4.63a	3.32	0.49	0.616	0	–
Chicken roast	1.85a	1.68	2.24ab	2.20	2.49b	2.46	5.51	0.004	0.02	0.31
Junk food	4.40a	3.12	4.42a	3.03	5.39b	3.41	7.07	0.001	0.02	0.31/0.30
Movie and popcorn	2.97a	2.63	3.07ab	2.70	3.60b	3.02	3.31	0.037	0.01	0.22
Obese and fries	5.62a	3.34	5.84a	3.31	6.06a	3.42	0.99	0.370	0	–
Mixed salad	2.06a	1.98	2.26a	2.26	2.34 a	2.32	1.02	0.360	0	–


*1 = “Not felt” and 10 = “Felt very strongly.” Means with differing letters within rows are significantly different at the *P* < 0.05 based on Tukey’s HSD paired comparisons. ^∗^Cohen’s *d* values are calculated between the significantly different means; when two values are shown, the first corresponds to the comparison evening meal vs. overeating, and the second one to the comparison positive meal vs. overeating. Sample sizes of conditions: evening meal *N* = 253; positive meal *N* = 247; overeating occasion *N* = 210.*

H2b stated that the overeating condition would elicit particularly higher *shame* and *guilt* when evaluating images depicting high-caloric foods or someone eating these foods than the other two conditions. When inspecting *guilt* and *shame* ratings, the same two general results emerge. As seen for the average negative score, people in the overeating occasion condition evaluated the image “obese and junk food” with the highest *guilt* and *shame* ratings. However, the score of *shame* was only significantly different from that obtained in the evening meal (*M*_O_ = 6.51, *SD*_O_ = 3.31 vs. *M*_E_ = 5.44, *SD*_E_ = 3.41, *P* = 0.002, *d* = 0.32), and the score of *guilt* did not significantly differ in the three conditions. Regarding the evaluation of the image “junk food,” people in the overeating condition scored significantly higher for both *shame* and *guilt* than those in the other two conditions (**Table 3**). This effect was also seen for *shame* in the image “movie and popcorn,” whereas the average of *guilt* in the overeating condition only significantly differed from that in the evening meal condition (*M*_O_ = 3.60, *SD*_O_ = 3.02 vs. *M*_E_ = 2.97, *SD*_E_ = 2.63, *P* = 0.041, *d* = 0.22). Therefore, H2b was completely supported for “junk food,” but for the data of “obese and junk food” it was only partly supported. Interestingly, H2b could also be supported for the “movie and popcorn” image for the *shame* ratings and partly for the *guilt* ratings.

### Moderating Effect of Dietary Restraint on Image Evaluation of Shame and Guilt Following Memory Priming

Further to understanding how mood manipulation through memory priming influenced emotional associations to the food and food–behavior images, H3 dictated that analyses be performed to explore whether participants’ level of dietary restraint (classified as “high” or “low”) exerted an influence on responses of shame and guilt.

H3a stated that highly restrained participants would associate stronger *guilt* and *shame* to the unhealthiest food-related images than unrestrained participants, and H3b specified that this difference would be larger in the overeating memory condition. Dietary restraint had a main effect on the two emotions when people evaluated the images “junk food” and “obese and junk food” (**Table 4**). Higher restraint led to the highest ratings of these two images. For “junk food” the *shame* ratings were: *M*_H_ = 4.22, *SD*_H_ = 3.12 vs. *M*_L_ = 3.34, *SD*_L_ = 2.89, *P* < 0.0001, *d* = 0.29, for “obese and junk food” *M*_H_ = 6.49, *SD*_H_ = 3.16 vs. *M*_L_ = 5.45, *SD*_L_ = 3.38, *P* < 0.0001, *d* = 0.31. Regarding the *guilt* ratings, for “junk food” these were: *M*_H_ = 5.26, *SD*_H_ = 3.27 vs. *M*_L_ = 4.18, *SD*_L_ = 3.05, *P* < 0.0001, *d* = 0.34, and for “obese and junk food” *M*_H_ = 6.30, *SD*_H_ = 3.24 vs. *M*_L_ = 5.39, *SD*_L_ = 3.39, *P* < 0.0001, *d* = 0.27. We therefore see an effect of restraint on guilt and shame on, particularly, images that were related to unhealthy food or food-related behavior. Therefore, H3a is confirmed.

**Table 4:** Results pertaining to Hypothesis 3. ANOVA results for emotions shame and guilt considering dietary restraint, memory condition, and their interaction as independent variables.

Image/Source		Shame			Guilt		
			
	*df*	*F*	*P*	*η*^2^	*F*	*P*	η^2^
**Burnt food**
Restraint	1,704	2.72	0.100	–	5.13	0.024	0.01
Condition	2,704	0.22	0.805	–	0.35	0.708	–
Restraint × Condition	2,704	2.28	0.104	–	0.64	0.529	–
**Chicken roast**
Restraint	1,704	0.03	0.853	–	0.07	0.789	–
Condition	2,704	1.67	0.190	–	5.53	0.004	0.02
Restraint × Condition	2,704	0.21	0.813	–	0.95	0.387	–
**Junk food**
Restraint	1,704	13.21	0.000	0.02	18.32	0.0001	0.02
Condition	2,704	5.51	0.004	0.01	5.92	0.003	0.02
Restraint × condition	2,704	4.42	0.012	0.01	3.09	0.046	0.01
**Movie and popcorn**
Restraint	1,704	0.68	0.408	–	4.92	0.027	0.01
Condition	2,704	6.34	0.002	0.02	2.86	0.058	–
Restraint × Condition	2,704	1.65	0.193	–	1.69	0.185	–
**Obese and junk food**
Restraint	1,704	15.99	0.0001	0.02	12.59	0.000	0.02
Condition	2,704	5.32	0.005	0.01	0.72	0.485	–
Restraint × Condition	2,704	1.34	0.261	–	0.95	0.388	–
**Mixed Salad**
Restraint	1,704	0.36	0.547	–	0.42	0.520	–
Condition	2,704	3.55	0.029	0.01	1.07	0.345	–
Restraint × Condition	2,704	0.44	0.642	–	0.30	0.739	–


*η^*2*^ effect sizes are only shown for the significant *F*s. Sample sizes of conditions: evening meal *N* = 253; positive meal *N* = 247; overeating occasion *N* = 210. Sample sizes of restraint groups: low restraint *N* = 371; high restraint *N* = 339.*

It was, however, only for the “junk food” image that a main effect of memory condition, as well as an interaction between dietary restraint level and memory condition, were observed for both *shame* and *guilt* (**Table 4**). These results are described next. Low restrained people reported feeling low levels of *shame* in the evening meal condition (*M*_LE_ = 2.59, *SD*_LE_ = 2.31), a score which was significantly different from those low restrained in the overeating condition (*M*_LO_ = 4.10, *SD*_LO_ = 3.33, *P* = 0.002, *d* = 0.53). In contrast, the high restrained group felt higher levels of *shame* both in the evening meal (*M*_HE_ = 4.29, *SD*_HE_ = 3.26) and in the overeating occasion (*M*_HO_ = 4.58, *SD*_HO_ = 3.18), though none of the three conditions significantly differed. Rejecting H3b, the largest difference in *shame* between the two restraint groups were only observed within the evening meal condition (*P* < 0.0001, *d* = 0.61), not in the overeating condition.

In the case of the “junk food” image, the data for *guilt* followed a similar pattern of results. Low restrained people in the evening meal condition reported feeling the lowest *guilt* feeling, followed by those in the positive meal condition and finally by those in the overeating condition (*M*_LE_ = 3.63, *SD*_LE_ = 2.78; *M*_LP_ = 4.30, *SD*_LP_ = 3.03; *M*_LO_ = 4.80, *SD*_LO_ = 3.31), though in this occasion the difference among the three conditions did not differ. Those highly restrained reported the higher *guilt* ratings under the evening meal (*M*_HE_ = 5.30, *SD*_HE_ = 3.26) and the overeating occasion (*M*_HO_ = 5.89, *SD*_HO_ = 3.41), which significantly differed from the ratings provided by those in the positive meal condition (*M*_HP_ = 4.56, *SD*_HP_ = 3.03; *P* = 0.022, *d* = 0.41). Also of relevance is the fact that the two restraint groups only differed in the evening meal (*P* < 0.0001, *d* = 0.55). Therefore, for *guilt* too, H3b was rejected since the interaction between memory condition and dietary restraint level was not in the expected direction.

## Discussion

The goal of this study was to investigate how memories linked to food can change our mood/emotional state, and how that emotional state, in turn, incidentally influences emotional judgements of food and food-related actions. Three different memory priming conditions were used (a positive meal, an evening meal, and having overeaten) with the expectation that participants would tend to evaluate the images of food/food-related actions in accordance with their prevailing emotional state as evoked by the memories. The results revealed several of the expected responses, mainly when related to negative emotions. Contrary to H1, a positive memory condition (the positive meal) did not lead to a significantly higher positive emotional evaluation of any of the images. Some evidence also suggests, however, that positive emotional states may be more effective than negative ones in influencing evaluations, although the extant literature on this relates mostly to social judgments (e.g., Forgas and Bower, 1987). What is more, other memory conditions led to a higher positive evaluation. In particular, the overeating condition led to a marginally higher positive evaluation for the “mixed salad” image. Although this was not part of our hypotheses, it can be explained in terms of a contrast effect. Contrast effect is likely to take place when the content of a target image is incongruent with the specific emotional state induced by the memory priming (Forgas, 1995). In this case, it could be possible that many people imagined a hearty meal and the image of a “healthier” food did not elicit a positive emotional state as otherwise. In the case of those in the overeating condition, an image of a “healthier” food seemed to have evoked more positive feelings or reappraise them, veiling their negative state (Saintives and Lunardo, 2016).

However, consistent with H2, the memory conditions, namely the overeating occasion, exerted a stronger impact on the negative emotional state of respondents, which was reflected in the negative emotion average score, and to a larger extent in *guilt* and *shame* ratings. In addition, as expected, these emotions were stronger in the evaluations of junk food or images depicting mindless eating behavior. Only the image “junk food,” where no person/actor was shown, lead to higher ratings of these two emotions from those in the overeating condition than from the other conditions. Moreover, *guilt* ratings were higher than *shame* ratings across the three conditions, which is reasonable considering that guilt is a negative emotion that is experienced when individuals appraise negative outcomes to their specific actions rather than viewing their entire self-negatively (Tracy and Robins, 2004; Blum, 2008). It should be noted that in the “movie and popcorn” image, showing the “self” (only arms and hands are shown) eating popcorn, *shame* and *guilt* follow a very similar pattern as with the image “junk food.” In contrast, for the “obese and junk food” image, which depicted an obese person, *shame* ratings were significantly higher in the overeating condition, and *guilt* did not differ between the three conditions. This could be reasoned by the fact that this image drew participants’ focus on the deficiency of their entire selves, mostly for those primed with the overeating occasion. This is in alignment with the notion that emotions systematically influence judgments by altering construal levels: *guilt* resulting in lower construal levels and *shame* leading to higher construal levels (Han et al., 2014). It is worth noting that the positive meal occasion exerted a close effect to that of the overeating occasion, possibly due to the fact that positive meals are often copious.

Second, the moderating effect of dietary restraint on the emotion evaluations was investigated (H3). We expected to find larger memory priming effects on the emotional evaluation of the images among participants who were more restrained. Dietary restraint moderated the effects in accordance with expectations. For instance, the evaluation of images depicting junk food items (“junk food” and “obese and junk food”) revealed that, on average, primarily the high restrained consumers in the overeating priming condition gave higher ratings of *guilt* and *shame*. While this finding was confirmed, what was unexpected was the finding that the ratings of highly restrained people in the evening meal condition would be as high.

The effects were dependent on the particular target images being assessed. Thus, it is plausible that the reported emotional response resulted from the combination of the memories that the images themselves triggered and those emotions recently activated by the memory primes, leading to an enhanced emotional response if these were in the same valence-arousal dimensions, a diminished one if in opposite directions, and altered in a different way if orthogonal. We observed that a dominantly positive memory (i.e., positive meal) and a more negative memory (i.e., having overeaten) did not modulate congruently (in a positive and negative way, respectively) the evaluation of the images, as would have been expected if the target images were human faces (Forgas et al., 1984; Forgas and Bower, 1987; Mojet et al., 2015). This can be explained by the fact that the images did not evoke one main emotion, but a combination of emotions. In addition, mood-priming is unlikely when subjects do not use substantive processing. For example, mood-incongruent (contrast) evaluation may occur because of the use of motivated processing in order to maintain or repair one’s mood (Erber and Erber, 1994; Sedikides, 1994) by selectively searching for contrasting rather than congruent information (Wyer and Srull, 1989). It seems this is the reason why for the “mixed salad” image, generally healthy and associated with positive emotions, participants in the overeating memory priming condition scored positive emotions higher and negative emotions lower relative to participants in the positive meal memory condition. Therefore, motivated processing can not only eliminate affect infusion, but can also produce mood-incongruent outcomes (Erber and Erber, 1994).

Finally, it was interesting to observe incidental effects from different personal past memories on evaluation of food-related images. The instructions given to participants in the memory task were relatively broad (e.g., “Try to recall as vividly as possible one of your usual weekday evening dinner occasions”), likely resulting in very different types of memory within each memory condition. Nonetheless, they seemed to be a powerful prime, even if the memory condition occurred as an earlier task without explicit connection to the emotion rating task. Although respondents were not asked at the end about their thoughts on the purpose of the questionnaire, they were asked about their general thoughts, and none mentioned the connection between the two tasks.

It is worth noting that although the positive meal memory was more vividly recalled (self-report scores), it was the memory of having overeaten that was associated with most significant effects. Apart from the fact that this was the most “negative” memory, this could also be due to the fact that the evoked memory was more emotionally constrained than the other two (“Try to recall as vividly as possible a past specific occasion after having mindlessly eaten much more than intended on your own”).

To conclude, the findings show evidence of the emotional impact that personal food memories have on consumers and suggest that the approach may be tapping into possibly unconscious emotions toward foods and food-related behavior. In addition, the results also reflected the influence of dietary restraint. Accessing emotional information indirectly through people’s evaluation of foods and behavior, apart from obtaining direct self-reflective responses, could be a good predictor of future behavior or food choice. This research therefore extends current knowledge on memory influencing people’s enjoyment (Robinson et al., 2011, 2012), intake (Higgs, 2002; Vartanian et al., 2016), and eating attitudes about food by focusing on its affect load. During eating occasions, we are not necessarily aware of what we are experiencing, and even less reflecting on it (Berridge and Winkielman, 2003; Köster and Mojet, 2015). The current approach could therefore potentially be useful to explore implicit emotion as an emotional state that expresses itself in experiences, thoughts or actions without conscious awareness of that state by the person.

## Author Contributions

BP-F and SJ were equally involved in the conception of the work, analysis, and interpretation of data for the work, the drafting the manuscript, and the final approval of the version to be published.

## Conflict of Interest Statement

The authors declare that the research was conducted in the absence of any commercial or financial relationships that could be construed as a potential conflict of interest.
