# Supplementary material for: The Incidental Influence of Memories of Past Eating Occasions on Consumers’ Emotional Responses to Food and Food-Related Behaviors
Source: Front Psychol. 2016 Jun 21;7:943. doi: 10.3389/fpsyg.2016.00943 (PMC4914557; doi:10.3389/fpsyg.2016.00943)
Supplement: Supplementary file 1 [file Table_1.DOCX]

Supplementary Material

The incidental influence of memories of past eating occasions on consumers’ emotional responses to food and food-related behaviours

Betina Piqueras-Fiszman *, Sara R. Jaeger

*** Correspondence:** Corresponding Author: betina.piquerasfiszman@wur.nl

**Table S1.** Mean ratings of the emotions, arousal, and valence obtained from the pre-tests of the 28 images selected for the main study. Emotion ratings were collected on a 1-10 Bullseye scale, and arousal and valence on a 1-9 SAM scale.

| Image | Enthusiasm | Joy | Happiness | Content | Surprise | Boredom | Relax | Frustration | Disappoint. | Worry | Sadness | Arousal | Valence |
| --- | --- | --- | --- | --- | --- | --- | --- | --- | --- | --- | --- | --- | --- |
| Burnt food (T) | 2.4 | 2.3 | 2.5 | 2.5 | 3.7 | 2.8 | 2.6 | 6.4 | 7.1 | 4.6 | 5.6 | 6.2 | 1.7 |
| Junk food (T) | 4.1 | 4.6 | 4.8 | 4.8 | 3.2 | 3.3 | 5.2 | 2.8 | 2.8 | 2.9 | 2.5 | 5.4 | 4.3 |
| Movie & Popcorn (T) | 4.3 | 5.2 | 5.7 | 6.2 | 3.2 | 3.5 | 7.4 | 2.3 | 2.2 | 2.0 | 2.1 | 5.7 | 4.7 |
| Roast Chicken (T) | 5.5 | 6.3 | 6.8 | 6.7 | 3.8 | 2.1 | 5.8 | 1.7 | 1.7 | 1.6 | 1.8 | 4.8 | 5.9 |
| Mixed Salad (T) | 5.0 | 5.3 | 5.6 | 5.6 | 3.6 | 3.1 | 5.5 | 2.3 | 2.7 | 2.2 | 2.1 | 5.8 | 5.1 |
| Obese& Junk food (T) | 2.4 | 2.7 | 2.9 | 3.6 | 2.8 | 5.5 | 4.3 | 5.3 | 5.9 | 5.7 | 5.6 | 6.3 | 2.2 |
| Coiled snake (F) | 2.5 | 2.2 | 2.3 | 2.3 | 5.5 | 2.4 | 2.1 | 3.0 | 2.7 | 6.8 | 3.1 | 5.0 | 2.2 |
| Oldies biking (F) | 6.5 | 7.0 | 8.1 | 7.1 | 3.5 | 2.2 | 7.3 | 2.0 | 1.8 | 1.8 | 1.8 | 5.7 | 5.5 |
| Toddler & Puppy (F) | 6.4 | 7.7 | 8.1 | 6.9 | 4.0 | 2.3 | 6.4 | 1.8 | 1.8 | 1.9 | 1.6 | 5.3 | 6.2 |
| Rain& Traffic (F) | 2.1 | 2.0 | 2.3 | 2.2 | 2.6 | 6.1 | 2.2 | 6.8 | 6.1 | 4.8 | 5.0 | 6.1 | 2.1 |
| Fresh air | 6.7 | 7.8 | 8.1 | 7.1 | 3.5 | 1.9 | 7.5 | 1.7 | 1.8 | 1.6 | 1.7 | 5.5 | 6.1 |
| Runners in coast | 5.2 | 6.7 | 7.4 | 7.5 | 3.8 | 2.3 | 8.4 | 1.8 | 1.8 | 1.8 | 1.9 | 5.7 | 6.1 |
| Candlelight dinner | 5.7 | 6.8 | 7.5 | 6.8 | 5.1 | 2.1 | 7.4 | 1.9 | 1.9 | 1.9 | 1.9 | 5.3 | 5.7 |
| Rollercoaster | 7.2 | 7.8 | 7.7 | 5.2 | 5.8 | 2.0 | 4.3 | 2.0 | 1.7 | 2.8 | 1.9 | 4.1 | 5.5 |
| Children | 6.7 | 6.8 | 7.0 | 6.3 | 3.8 | 2.3 | 5.8 | 2.0 | 2.0 | 2.0 | 1.8 | 5.5 | 5.2 |
| Running | 5.8 | 5.0 | 5.3 | 4.8 | 3.4 | 2.9 | 5.0 | 2.3 | 2.2 | 2.3 | 2.1 | 5.5 | 4.4 |
| Blue sky | 5.1 | 6.7 | 7.2 | 6.7 | 3.7 | 2.4 | 7.5 | 2.0 | 2.0 | 1.8 | 1.9 | 6.0 | 5.5 |
| Neighbors | 6.2 | 7.2 | 7.7 | 6.8 | 4.2 | 2.1 | 6.5 | 1.8 | 2.0 | 1.8 | 1.8 | 5.6 | 5.5 |
| Flower bouquet | 4.9 | 6.5 | 6.7 | 5.8 | 5.6 | 2.5 | 5.3 | 1.8 | 2.0 | 1.7 | 2.0 | 5.6 | 5.5 |
| Yoga | 5.9 | 6.3 | 6.9 | 6.0 | 3.8 | 2.8 | 6.2 | 1.9 | 1.9 | 2.2 | 1.9 | 5.8 | 5 |
| Contamination | 2.0 | 2.3 | 2.3 | 2.3 | 2.8 | 4.2 | 2.3 | 6.1 | 5.6 | 5.9 | 5.6 | 6.0 | 1.8 |
| Cemetery | 1.8 | 2.0 | 2.1 | 2.2 | 2.3 | 2.7 | 2.8 | 3.1 | 4.2 | 4.9 | 7.9 | 6.6 | 1.7 |
| Eating & Working | 2.7 | 2.6 | 2.9 | 3.0 | 2.7 | 4.6 | 3.2 | 4.6 | 3.8 | 4.6 | 3.4 | 6.0 | 2.9 |
| Lazy kids | 2.5 | 2.9 | 3.6 | 4.0 | 3.2 | 5.7 | 4.9 | 5.0 | 5.2 | 5.1 | 4.7 | 5.9 | 2.7 |
| Pamper | 5.0 | 6.3 | 6.8 | 6.0 | 6.0 | 2.2 | 5.3 | 2.0 | 2.0 | 2.0 | 1.9 | 5.5 | 5.2 |
| Burning forest | 2.0 | 1.8 | 1.8 | 2.0 | 4.5 | 2.1 | 1.7 | 6.0 | 6.1 | 8.1 | 7.8 | 4.7 | 1.1 |
| Kids fighting | 2.4 | 2.0 | 2.0 | 2.0 | 3.4 | 2.9 | 1.9 | 6.4 | 6.2 | 7.2 | 7.0 | 5.3 | 1.4 |
| Burglary | 1.8 | 1.6 | 1.6 | 1.9 | 4.1 | 2.5 | 1.6 | 6.7 | 7.2 | 8.0 | 6.7 | 5.2 | 1 |

Note: (F) denotes the image is a filler, and (t) denotes the image is a target.
