# Supplementary material for: The Incidental Influence of Memories of Past Eating Occasions on Consumers’ Emotional Responses to Food and Food-Related Behaviors
Source: Front Psychol. 2016 Jun 21;7:943. doi: 10.3389/fpsyg.2016.00943 (PMC4914557; doi:10.3389/fpsyg.2016.00943)
Supplement: Supplementary file 2 [file Table_2.DOCX]

Supplementary Material

The incidental influence of memories of past eating occasions on consumers’ emotional responses to food and food-related behaviours

Betina Piqueras-Fiszman *, Sara R. Jaeger

*** Correspondence:** Corresponding Author: betina.piquerasfiszman@wur.nl

**Table S2.** Results from the manipulation check, showing for each of 18 emotion words the mean ratings evoked by the images when participants (*N* = 401) did not complete a memory conditioning task. Emotion responses obtained using a bulls-eye approach where 1= “the picture does not convey this feeling” and 10= “the picture strongly conveys this feeling”.

| Emotion terms | Junk Food | Obese & Junk Food | Mixed Salad | Movie & Popcorn | Burnt Food | Roast Chicken | Traffic & Rain | Coiled Snake | Oldies Biking | Toddler & Puppy | F(9,4000) | *p* | η^2^ |
| --- | --- | --- | --- | --- | --- | --- | --- | --- | --- | --- | --- | --- | --- |
| Relax | 5.2c | 4.2b | 5.7cd | 7.3f | 2.7a | 6.2de | 2.4a | 2.3a | 6.7ef | 6.4e | 175.9. | < .001 | .28 |
| Content | 5.0c | 3.5b | 5.6cd | 6.2de | 2.7a | 6.6e | 2.7a | 2.4a | 6.3e | 6.7e | 151.99 | < .001 | .25 |
| Happiness | 5.5b | 3.2a | 6.0b | 6.1b | 2.7a | 6.9c | 2.7a | 2.6a | 7.2cd | 7.8d | 213.26 | < .001 | .32 |
| Warm-heartedness | 4.4b | 2.8a | 4.9bc | 5.2c | 2.6a | 6.4d | 2.5a | 2.4a | 6.5d | 7.8e | 199.46 | < .001 | .31 |
| Joy | 4.9b | 2.8a | 5.5b | 5.5b | 2.5a | 6.5c | 2.4a | 2.6a | 6.5c | 7.3d | 178.31 | < .001 | .29 |
| Enthusiasm | 4.2b | 2.5a | 5.0cd | 4.4bc | 2.6a | 5.5de | 2.5a | 2.6a | 5.9e | 5.9e | 103.06 | < .001 | .19 |
| Pride | 3.1c | 2.5ab | 4.4d | 3.1bc | 2.4a | 5.0e | 2.2a | 2.6abc | 5.0de | 5.1e | 72.49 | < .001 | .14 |
| Nostalgia | 3.6c | 2.8ab | 3.3bc | 4.4d | 2.8ab | 5.3e | 2.8ab | 2.4a | 5.2e | 5.2e | 62.31 | < .001 | .12 |
| Courage | 2.7abc | 2.2a | 3.1cde | 2.6abc | 2.5ab | 3.0bcd | 2.9bc | 4.1f | 3.6ef | 3.6def | 20.09 | < .001 | .04 |
| Surprise | 3.4abcd | 2.8a | 3.6bcd | 3.1ab | 4.0d | 3.5bcd | 2.8a | 5.3e | 3.4abc | 3.9cd | 25.98 | < .001 | .06 |
| Boredom | 3.2c | 4.6d | 2.8bc | 3.0c | 3.0c | 2.2ab | 5.4e | 2.1a | 2.2ab | 2.1a | 76.83 | < .001 | .15 |
| Worry | 3.1b | 5.0c | 2.1a | 2.2a | 4.7c | 2.2a | 4.9c | 6.3d | 2.0a | 2.2a | 154.07 | < .001 | .26 |
| Frustration | 2.8bc | 4.3d | 2.3ab | 2.3ab | 6.1e | 2.2ab | 6.6e | 3.1c | 2.1a | 1.9a | 175.30 | < .001 | .28 |
| Tension | 2.6a | 3.7b | 2.1a | 2.2a | 4.7c | 2.2a | 5.8d | 6.6e | 2.1a | 2.0a | 192.00 | < .001 | .30 |
| Sadness | 2.8bc | 5.2de | 2.3ab | 2.3ab | 5.3e | 2.3ab | 4.7d | 3.0c | 2.1a | 2.1a | 108.33 | < .001 | .20 |
| Disappointment | 3.0d | 5.1e | 2.5bcd | 2.3abc | 6.4f | 2.2ab | 5.5e | 2.9cd | 2.1aab | 1.9a | 159.35 | < .001 | .26 |
| Guilt | 4.6c | 5.5d | 2.2a | 2.9b | 4.3c | 2.4ab | 2.3ab | 2.3a | 2.0a | 1.8a | 108.17 | < .001 | .20 |
| Shame | 3.5c | 5.5e | 2.0ab | 2.4ab | 4.8d | 2.1ab | 2.5b | 2.2ab | 2.0ab | 1.9a | 116.93 | < .001 | .21 |

Note: Means with differing subscripts within rows are significantly different at the p < .05 based on Tukey’s HSD paired comparisons.
