# Supplementary material for: The Incidental Influence of Memories of Past Eating Occasions on Consumers’ Emotional Responses to Food and Food-Related Behaviors
Source: Front Psychol. 2016 Jun 21;7:943. doi: 10.3389/fpsyg.2016.00943 (PMC4914557; doi:10.3389/fpsyg.2016.00943)
Supplement: Supplementary file 3 [file Image_1.PDF]

Supplementary Material

The incidental influence of memories of past eating occasions on consumers' emotional responses to food and food-related behaviours

Betina Piqueras-Fiszman *, Sara R. Jaeger

*** Correspondence:** Corresponding Author: betina.piquerasfiszman@wur.nl

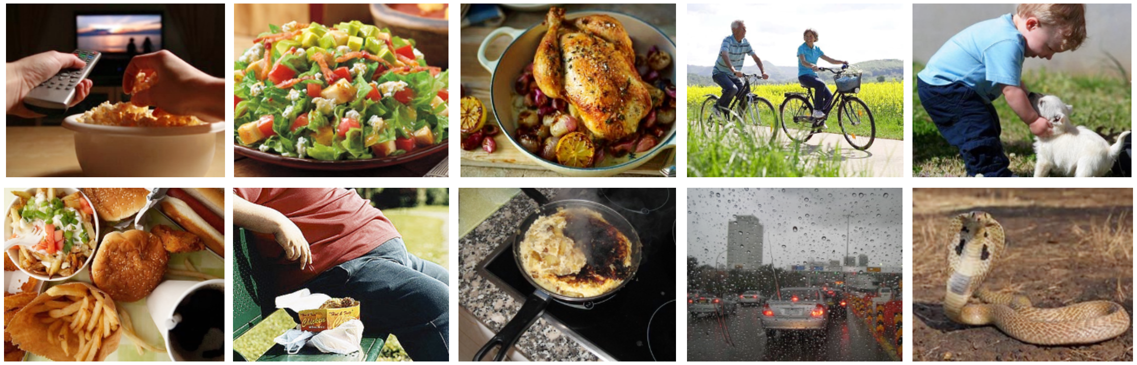

Supplementary Figure 1. The 10 images used as stimuli. The three columns on the left are the six target images and the last two columns are the four filler images.
